# Endoscopic Vacuum Therapy of Upper Gastrointestinal Anastomotic Leaks: How to Deal with the Challenges (with Video)

**DOI:** 10.3390/life13061412

**Published:** 2023-06-19

**Authors:** Laurent Monino, Tom G. Moreels

**Affiliations:** Department of Gastroenterology & Hepatology, Cliniques Universitaires Saint-Luc, Avenue Hippocrate 10, B-1200 Brussels, Belgium

**Keywords:** upper gastrointestinal tract, anastomotic leak, endoscopic vacuum therapy, practical review

## Abstract

Anastomotic leaks after gastrointestinal surgery have an important impact on surgical outcomes because of the high morbidity and mortality rates. Multiple treatment options exist requiring an individualized patient-tailored treatment plan after multidisciplinary discussion. Endoscopic vacuum therapy (EVT) is a novel treatment option that is nowadays recognized as an effective and useful endoscopic approach to treat leaks or perforations in both the upper and lower gastrointestinal tract. EVT has a very good safety profile. However, it is a time-consuming endeavour requiring engagement from the endoscopist and understanding from the patient. To the unexperienced, the EVT technique may be prone to several hurdles which may deter endoscopists from using it and depriving patients from a potentially life-saving therapeutic option. The current review highlights the possible difficulties of the EVT procedure and aims to provide some practical solutions to facilitate its use in daily clinical practice. Personal tips and tricks are shared to overcome the pre-, intra- and post-procedural hurdles. An instructive video of the procedure helps to illustrate the technique of EVT.

## 1. Introduction

Upper gastrointestinal tract surgery for both benign and malignant indications is prone to postoperative adverse events, with anastomotic leakage being one of the most challenging to treat. The risk and the location of the anastomotic leak depends on the type of surgical intervention and different classifications of anastomotic leaks exist [1,2]. The site of the leak and the extent of the corresponding extraluminal collection depends on the type of surgical intervention [2]. Despite new developments such as minimally invasive and robotic-assisted surgical techniques, anastomotic leak remains a frequent adverse event, occurring in up to one out of three patients and usually appearing in the early postoperative period [3,4,5]. Leaks may vary from a minor anastomotic fistula to complete dehiscence of the anastomosis, greatly impacting the surgical outcome, with high morbidity and mortality up to 25% [5,6,7]. Apart from the major clinical implications, postoperative anastomotic leaks after oesophageal surgery also have an important economic impact, nearly doubling the amount of the admission cost compared to non-complicated surgery [8]. A patient-tailored approach is required, and different therapeutic options are currently available, ranging from redo surgery, interventional radiology and endoscopy, often with a combination of techniques, depending on local availability and expertise, but also on size and type of the defect [9,10]. None of these rescue techniques is currently superior over the other ones. Therefore, possible therapeutic options should be discussed on a multidisciplinary base with the surgeons, radiologists and endoscopists to optimize the treatment plan [11]. The patient should be informed about the duration of the treatment, the potential risks and the expected outcome.

With the advent of new techniques and therapeutic options, endoscopy has become an important player in the treatment of anastomotic leaks in the upper gastrointestinal tract [12]. The endoscopic approach has been reviewed recently and encompasses internal drainage techniques of extramural collections using double-pigtail stents and closure techniques of the anastomotic defect using covered metallic stents, through-the-scope or over-the-scope clips, and endoluminal suturing [9,12,13,14]. A newly adopted option is endoscopic vacuum therapy (EVT), which has been shown to be very effective in draining extramural collections, reducing their size to even complete closure of the anastomotic defect, or improving the inflammatory tissue environment before redo surgery [15,16,17,18]. Prospective randomized comparative studies are currently missing due to the often life-threatening condition of the patients with postoperative leaks, requiring an individualized patient-tailored approach. Research data are therefore based on retrospective case series, with a possible selection bias when trying to compare the efficacy and safety of the different available techniques. EVT is currently accepted as a valuable endoscopic treatment option of large-sized anastomotic leaks in both the upper and the lower gastrointestinal tract and appears to be more effective and less burdened by adverse events as compared to metallic stenting according to a recent meta-analysis published in this journal [19,20]. It was also shown to be useful for the treatment of duodenal perforations and defects after biliopancreatic surgery [12].

## 2. Literature Overview of EVT in the Upper Gastrointestinal Tract

The principle of EVT is based on continuous negative pressure applied to the walls of the extramural collection with an open-pore sponge, resulting in local arteriolar dilation, the promotion of granulation, and consecutive wound healing leading to fistula closure [21]. Although the beneficial application of wound negative-pressure suction dates back to the 1960s, the endoscopic use of vacuum therapy to treat anastomotic leaks was first reported in 2008 [22,23]. Table 1 provides an historical overview of the literature of EVT in the upper gastrointestinal tract. Despite the fact that the available research data are based on retrospective case series, prone to selection bias, Table 1 clearly demonstrates that EVT reaches very high success rates (85% in the largest case series including 156 patients) to treat postoperative leaks and perforations in the upper gastrointestinal tract [24]. EVT turns out to be more effective in fistula closure and in the reduction in leak-related mortality than the use of fully covered metal stents, which was long considered the standard endoscopic approach of anastomotic leak closure [16,17,20]. Prospective randomized trials comparing EVT and stenting are now initiated, and the results are awaited [19]. According to the currently available data reviewed in a recent meta-analysis, EVT may lead to a paradigm shift in the treatment of large-sized postoperative anastomotic leaks in the upper gastrointestinal tract, being more efficacious than stenting [20]. However, Table 1 also shows that the mortality risk is still present in this group of critically ill patients under EVT treatment, the risk relating to both the underlying clinical condition and to the endoscopic therapy itself. This warrants continuous critical evaluation of the EVT indication before and during the therapy. In addition to the treatment of postoperative leaks in the upper gastrointestinal tract, EVT was shown to be effective to treat spontaneous and iatrogenic perforations in the upper gastrointestinal tract, including the duodenum (Table 1). Additionally, postoperative leaks after biliopancreatic, bariatric and colorectal surgery have been treated successfully using EVT [9,12,15,16,17,18,25,26]. A few retrospective studies indicate the beneficial effects of the combined use of EVT and stenting as a rescue technique for complex uncontained leaks in the upper gastrointestinal tract [27,28].

Despite the fact that EVT has been in use now for almost 15 years and with very good clinical results, it remains a challenging multi-step procedure with possible difficulties at different levels, hampering its use in daily clinical practice. The current review focusses on these difficulties and highlights the hurdles of EVT to treat anastomotic leaks in the upper gastrointestinal tract. Ready-to-use practical solutions for these caveats are provided where possible.

## 3. Endoscopic Vacuum Therapy Principle

The principle of EVT relies on the well-known negative-pressure therapy for external wound healing by secondary intention (per secundam) [21,74]. The negative pressure stimulates local tissue perfusion while decreasing tissue oedema. Continuous aspiration of secretions, pus and necrotic debris cleans the wound surface and stimulates vital granulation tissue, rendering the local environment healthier, ultimately leading to secondary wound healing. When transposing this principle of negative-pressure therapy for internal wound healing, such as an anastomotic leak after digestive surgery, endoscopy comes into play. The endoscope facilitates access to the fistula and the extramural collection to apply the negative-pressure therapy. Endoscopy also allows us to evaluate the size of the leak and the extension of the collection, as well as perform endoscopic debridement of necrotic tissue. To apply internal EVT, a flexible perforated drainage tube loaded with an open-pore element (sponge or compress) is placed endoscopically within the extramural cavity through the fistula and connected to a vacuum source [75]. With the active aspiration of necrotic debris and pus into the open-pore element, the cavity walls collapse, and the pores of the sponge become gradually saturated, resulting in less effective negative-pressure application. The success of the technique relies on the continuous negative-pressure suction on the cavity walls. Therefore, the drainage tube with the attached sponge needs to be replaced every 3 to 5 days. Since negative-pressure therapy results in a slow healing process per secundam, the total treatment duration usually takes several weeks, with a mean of 3 to 7 sponge exchange procedures, along with an endoscopic evaluation of the progressive reduction in cavity volume [15].

## 4. Endoscopic Vacuum Therapy Procedure

Video S1 shows the procedure of EVT in a patient with an anastomotic leak after oncological oesophagectomy. Under general anaesthesia with endotracheal intubation, diagnostic upper gastrointestinal endoscopy is performed to evaluate the surgical anastomosis, the location and size of the leak and the extent of the extramural cavity. Therapeutic endoscopy should always be performed using carbon dioxide insufflation to reduce luminal and extraluminal distension and to reduce the risk of air embolism in case of perforation [76]. Endoscopic debridement of necrotic tissue is performed whenever possible in order to optimize local healing conditions and to facilitate the optimal use of EVT. Next, the flexible overtube is placed over the endoscope outside the patient, and the overtube-loaded endoscope is reintroduced into the oesophagus and the cavity with or without fluoroscopic guidance. The overtube is gently pushed forward into the cavity using the endoscope as guidance. The endoscope is removed from the overtube, and the sponge-loaded drainage tube is pushed through the overtube into the cavity. The overtube is removed, leaving the sponge inside the cavity. The draining tube is then repositioned from the mouth into the nose with the rendezvous technique and fixed behind the ear like a conventional nasogastric tube. Endoscopic control of the correct position of the sponge inside the cavity may be warranted. Finally, the drainage tube is connected to the vacuum source, which, in general, is an electronic pump with a negative pressure of −75 mm Hg to −200 mm Hg. A vacuum redon drain or bottle can be used as an alternative for the electronic pump.

## 5. Difficulties of Endoscopic Vacuum Therapy in the Upper Gastrointestinal Tract

Despite the fact that EVT is nowadays a standardized procedure, it remains an enterprise prone to several hurdles and difficulties. They can be encountered in the pre-, intra- and post-procedure period, and also during the removal of the sponge and drainage tube.

### 5.1. Pre-Procedure Difficulties

**Figure 1:**
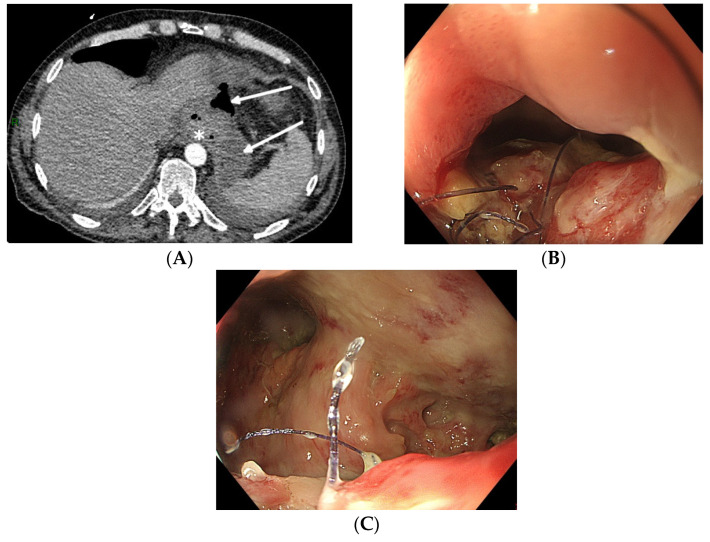
Total gastrectomy with an oesophagojejunal anastomosis complicated by an anastomotic leak. (**A**) Pre-procedure evaluation by CT scan (white arrows: hydroaeric collection; white star: anastomotic leak). (**B**) Pre-procedure endoscopic evaluation with inspection of the anastomotic leak. (**C**) Pre-procedure endoscopic evaluation of the extramural cavity.

Not all oesophageal perforations can be treated using EVT [77]. The technique relies on the collapse of the extramural collection under negative pressure. The presence of an oesophageal fistula to the respiratory system does not permit the necessary build-up of negative pressure, thus rendering EVT unsuccessful. Therefore, EVT should not be used to treat broncho-oesophageal fistulas, although there are a few case reports of successful EVT treatment [42]. Secondly, since the device stimulates local tissue perfusion, it is considered contra-indicated in a malignant environment because of the risk of tumour growth induction. Finally, complete anastomotic dehiscence due to ischemic conduit necrosis necessitates redo surgery, and EVT should not be attempted in these particular situations (beyond EVT). Pre-procedure evaluation with CT scan (with oral contrast) and upper gastrointestinal endoscopy is mandatory to correctly evaluate the indication for EVT and to facilitate a patient-tailored multidisciplinary treatment plan (Figure 1).

Although ready-to-use sets are commercially available (Endo-Sponge, Eso-Sponge; B. Braun Melsungen AG; Melsungen, Germany), their availability is not widespread, and their financial cost is significant. However, since the design of the device is not very complicated, it can be self-assembled by attaching an open-pore drainage film or a size-cut polyurethane sponge to a nasogastric tube with multiple side-holes around the distal tip [78,79]. It can be introduced into the cavity through the commercially available overtube, or it can be endoscopically manoeuvred using rat tooth forceps or a polypectomy snare, taking care not to damage the sponge during the introduction. In the absence of an electronic vacuum pump with alarm function, a vacuum redon drain or bottle can be used as a continuous vacuum source. However, neither the redon drain nor bottle allows us to pre-set the vacuum force precisely (high–medium–low), and regular evaluation of the vacuum force is mandatory when using these alternative vacuum sources. Whenever the vacuum suction force drops because the redon drain is filled with aspirated fluids and debris, it should be replaced immediately. The loss of continuous vacuum aspiration negatively impacts EVT efficacy.

### 5.2. Intra-Procedure Difficulties

**Figure 2:**
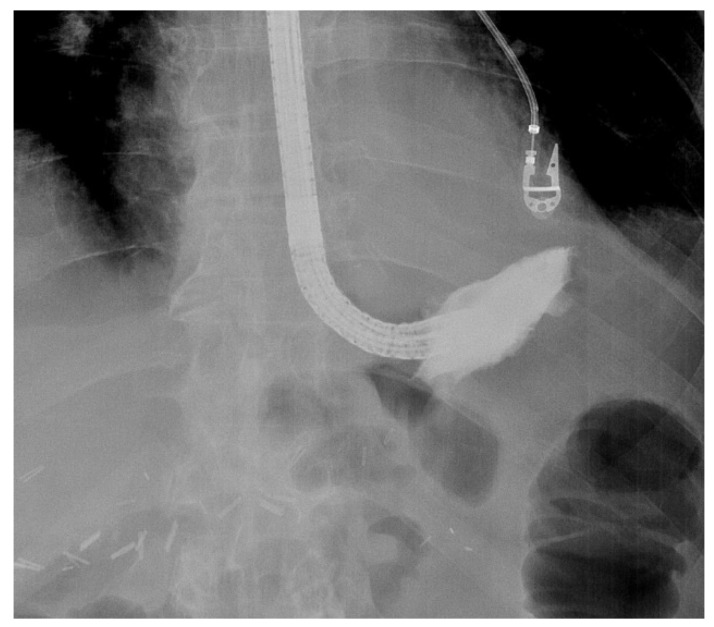
Fluoroscopic evaluation of the depth and the orientation of an extramural collection after endoscopic contrast injection in a patient with an anastomotic leak of an oesophagojejunal anastomosis.

When engaging into the EVT procedure of oesophageal anastomotic leaks, several difficulties can be encountered. Since this is a time-consuming procedure with multiple introductions of the endoscope, it is best performed under general anaesthesia with endotracheal intubation, to prevent secretions and debris from the collection entering the respiratory system and to improve working conditions for both the endoscopist as for the patient. Fluoroscopy may also help to evaluate the depth and the extent of the extramural collection at the beginning of the EVT treatment, without being mandatory for future sponge exchanges (Figure 2).

**Figure 3:**
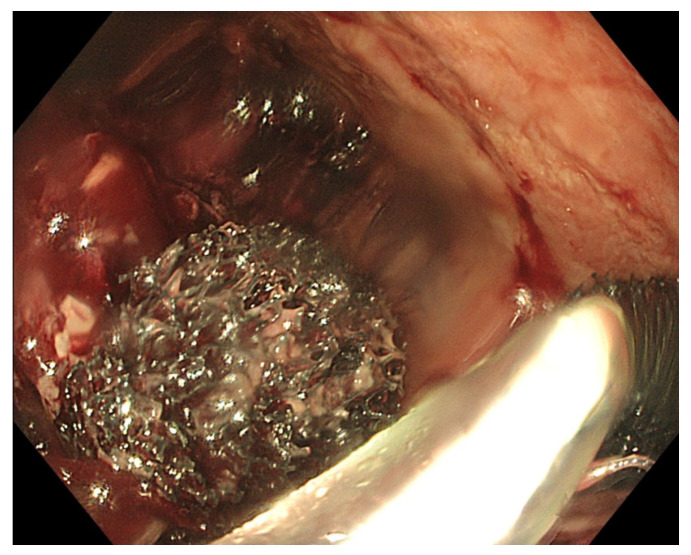
Placement of two sponges in a large-sized extramural collection (80 × 70 mm).

The size of the anastomotic fistula may range from a millimetric orificium to a nearly complete dehiscence of the anastomosis [7]. A small-sized fistula can be closed using clips or stents or endoscopic suturing. However, external drainage of an extramural collection is mandatory when closing the fistula. Internal drainage using double pigtail stents can be an option, as well as EVT. However, when the fistula diameter does not allow the introduction of the overtube and the sponge, balloon dilatation of the fistula tract is possible. Additionally, the size of the sponge can be adapted to the small volume cavity by cutting it to a smaller and compatible size. Finally, positioning of the sponge into the oesophageal lumen covering the fistula orificium is another option to treat small-sized fistula [80]. The latter is usually a less efficacious solution, since the majority of the negative-pressure force will be lost in the lumen of the gastrointestinal tract, and other closure techniques with clips or endoscopic suturing may be more appropriate in case of small-sized fistula [9]. Intraluminal positioning of the sponge can also be applied at the end of successful EVT treatment with collapse of the extramural cavity to close the remaining small-sized fistula tract. The intraluminal sponge placement provokes stasis of saliva above the sponge. As already mentioned, the size of the sponge can be adapted to the volume of a small collection by cutting the sponge to its correct size. Large-sized fistulas and collections or near-complete anastomotic dehiscence may need the introduction of more than one sponge in order to optimize vacuum therapy and to promote the collapse of the collection (Figure 3).

**Figure 4:**
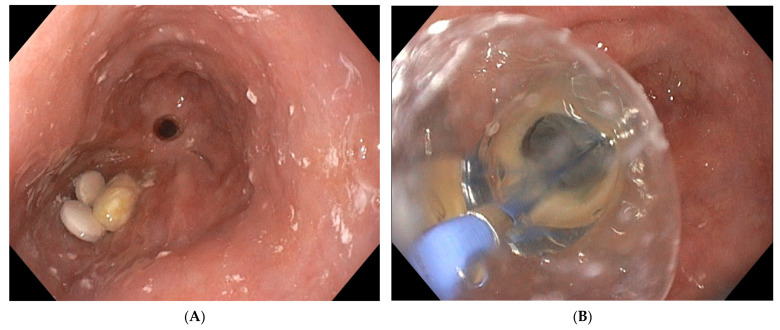
(**A**) Oesophageal stricture 6 months after intraluminal EVT treatment of partial anastomotic dehiscence. Notice the presence of medication tablets proximal to the post-EVT anastomotic stricture. (**B**) Endoscopic balloon dilatation of a post-EVT oesophageal stricture.

When opting for more than one EVT device, each device should be connected to its proper vacuum source to avoid the loss of aspiration through communicating vessels. In case of anastomotic dehiscence without extraluminal collection, the sponge should be positioned in the anastomosis maintaining a local negative pressure of −75 mm Hg to −125 mm Hg. However, this approach can induce an anastomotic fibrotic stricture as a result of successful EVT (Figure 4).

Large collections may contain necrotic debris, pus and fibrine. To avoid early saturation of the sponge with necrotic debris and a loss of negative-pressure force, the collection should be cleaned as much as possible before positioning the sponge. This can be performed via removal of the debris using rat tooth forceps, a polypectomy snare or mere rinsing and aspiration through the working channel of the endoscope. This cleaning procedure is indicated at every new sponge exchange.

**Figure 5:**
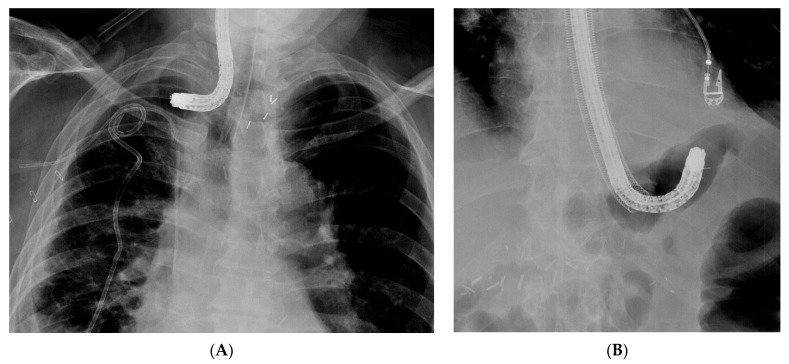
Difficult introduction of the overtube on the endosope into the collection. (**A**) Introduction of the endoscope into a collection, thus complicating an anastomotic oesophagogastric leak. The angulated tip of the endoscope does not allow advancement of the overtube into the collection. (**B**) Introduction of the overtube is not possible over the angulated endoscope tip in a patient with an anastomotic leak and extramural collection at the level of the oesophagojejunal anastomosis.

Successful sponge positioning relies on the correct introduction of the overtube into the collection. This can be challenging when the access to the collection requires an angulation of the tip of the endoscope (Figure 5).

Moreover, introducing the sponge through the overtube comes with a lot of friction, and it increases the risk of dislocation of the overtube from the collection into the oesophageal lumen. To reduce the friction, it is advised to use a silicon spray to lubricate the inner lumen of the overtube and to apply lubrification gel onto the sponge to facilitate its introduction through the overtube. Endoscopic control of the correct position of the sponge into the collection is warranted, since dislocation of both the overtube and the sponge is possible during the introduction process. In case of incorrect positioning of the sponge after removal of the overtube, the sponge should be removed, and the procedure should be restarted. As an alternative, the sponge can be endoscopically manoeuvred from the oesophageal lumen into the collection using rat tooth forceps, taking care not to damage the sponge while trying to reposition it. Additionally, in case of a self-assembled device lacking the overtube, the sponge-loaded tube can be taken by rat tooth forceps or a polypectomy snare before it is introduced endoscopically through the mouth and taken down into the oesophagus and the collection [78]. Correct positioning of the sponge into the collection or intraluminally adjacent to the fistula is mandatory for successful EVT. Migration of the sponge will negatively affect EVT efficacy. Endoscopic control of the correct position is therefore mandatory at the beginning and at the end of each sponge exchange procedure.

Although the manufacturer’s product information defines the position of the sponge-loaded flexible tube in the mouth, it is much more comfortable for the patient to position it through the nose like a classical nasogastric tube. Transnasal position of the tube will also avoid occlusion of the tube (and thus a loss of negative pressure) by biting it. The tube can be redirected from the mouth to the nose using the rendezvous technique with a naso-oral catheter. Transnasal position does not prevent the removal of the tube and the soft sponge through the nose, but this should only be performed under general anaesthesia with endotracheal intubation, in order to prevent aspiration of debris adherent to the sponge and to optimize the patient’s clinical comfort. The majority of patients undergoing EVT in the upper gastrointestinal tract are hospitalized until the end of the endoscopic treatment, but only a minority needs admission to the Intensive Care Unit.

### 5.3. Post-Procedure Difficulties

After exteriorisation of the tube through the nose, gentle traction should be applied before fixation of the tube, in order to maintain the sponge in the correct position and to avoid distal migration of the sponge. As mentioned before, endoscopic control of the correct final sponge position is warranted.

Connecting the vacuum source to the aspiration tube requires adjusted connectors depending on the type of vacuum source. As for the EVT device, a compatible electronic vacuum pump is not always available, and it can be replaced by a surgical vacuum redon drain or a vacuum bottle. These non-electronic vacuum sources usually allow high–medium–low aspiration force through a physical switch, in contrast to the electronic pump with an adjustable negative force up to −200 mm Hg. The use of a vacuum redon drain or bottle requires frequent evaluation of the negative pressure since there is no electronic alarm when the negative pressure drops too low. When the redon contains too much aspiration fluids from the collection, it should be replaced immediately in order to maintain effective negative pressure. Leaks at the connectors should also be looked for in case of a loss of negative pressure.

As mentioned before, EVT requires multiple replacements of the device in order to maintain a continuous negative pressure at the level of the anastomotic leak. Therefore, the sponge should be replaced every 3 to 4 days. A delay in the replacement of the sponge may, on the one hand, lead to a loss of negative pressure and, on the other hand, to gradual tissue ingrowth into the open pores of the sponge. This may render sponge removal more difficult, introducing the risk of sponge rupture [29]. Since multiple sponge replacements are required (usually during a procedure under general anaesthesia), the necessary endoscopy time slots in the upcoming weeks should be reserved once EVT is initiated and the first sponge is put in place. Reserved time slots every Monday and Thursday or every Tuesday and Friday are very practical, in order to avoid scheduling replacements during the weekends.

**Figure 6:**
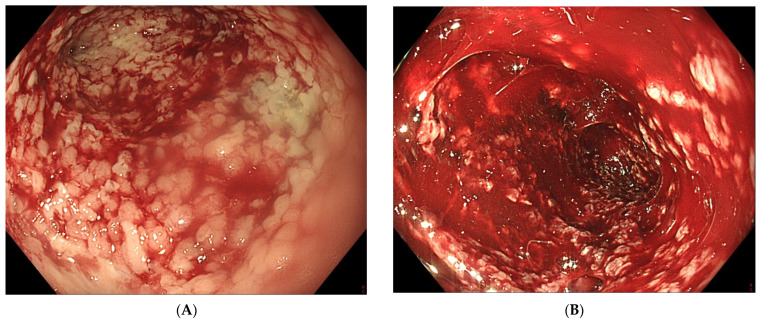
(**A**) Clean granular aspect of healing tissue in an extraluminal cavity after removal of the EVT sponge. (**B**) Diffuse intracavity bleeding after removal of the EVT sponge.

Transnasal removal of the sponge is feasible and safe, but should only be performed under general anaesthesia with endotracheal intubation to avoid aspiration of secretions, blood and debris in the respiratory system. Endoscopic evaluation of the correct position of the sponge is important before its removal to assure efficient EVT. It might be necessary to dislodge the sponge from the granulating tissue using the endoscope or rat tooth forceps to facilitate atraumatic dissection and removal. Endoscopic inspection of the residual cavity is not only mandatory to evaluate the reduction in its volume and the aspect of the granulation tissue, but also to exclude and remove retained fragments of the sponge, if any (Figure 6). Intracavitary bleeding may occur after sponge removal. It is usually diffuse oozing bleeding and represents a sign of favourable tissue healing (Figure 6). It generally stops after endoscopic rinsing and cleaning of the cavity. However, it is advised to stop anticoagulant therapy before removal of the sponge [77].

## 6. Adverse Events Relating to EVT in the Upper Gastrointestinal Tract

Patients requiring EVT to treat postoperative anastomotic leaks in the upper gastrointestinal tract are critically ill, presenting with a septic condition due to mediastinal infection with cardiorespiratory repercussion and a high mortality risk. They may require admission to the Intensive Care Unit. Despite the multiple EVT-related endoscopic interventions in these critically ill patients, the adverse event rate of EVT is low with mainly minor adverse events (Table 1). A recent meta-analysis reviewed the EVT-related adverse events in the upper gastrointestinal tract [81]. The overall mortality of patients undergoing EVT for oesophageal perforations or leaks was 7.1%, relating to the underlying septic condition, not to the EVT procedure itself. This mortality rate illustrates the severe clinical condition induced by an anastomotic leak in the upper gastrointestinal tract, rather than the EVT-related risks. However, a few cases of mortality directly relating to the EVT procedure itself have been described. Death was caused by uncontrollable bleeding after sponge removal due to the proximity of the thoracic aorta leading to a lethal aortic fistula [31,48]. Total EVT-related adverse event rate was 13.6%, characterized by EVT-induced anastomotic stricture (Figure 4), local bleeding after sponge removal (Figure 6) and sponge dislocation. Sponge rupture during removal can be avoided by respecting the foreseen time schedule of sponge replacement every 3 to 5 days. In general, EVT-related adverse events are minor and can be managed endoscopically (AGREE IIIa classification of endoscopic adverse events) [82]. On only very rare occasions, visceral injury such as spleen injury or bowel ischemia have been described as relating to the EVT procedure [83]. Despite the multiple endoscopic interventions in critically ill patients, EVT can be considered as a safe therapeutic procedure.

## 7. Conclusions

Anastomotic leaks after digestive surgery have an important impact on surgical outcome [5,6,7]. They represent a real therapeutic challenge because of the high morbidity and mortality rates. Multiple treatment options exist, often combining interventional radiology and endoscopy and even redo surgery [9,10]. To improve patient outcomes, it is of the utmost importance to provide an individualized patient-tailored treatment plan after multidisciplinary discussion [11]. EVT is nowadays recognized as an effective and useful endoscopic approach to treat leaks or perforations in both the upper and lower gastrointestinal tract. Moreover, it has become one of the most effective treatment options overall with a very good safety profile [20,81]. However, it is a time-consuming endeavour requiring engagement from the endoscopist and understanding from the patient. Correct information and communication about the outcome and expectations are mandatory. The current review highlights the possible difficulties of the technique of EVT and provides practical solutions.

Pre-procedural evaluation of the postoperative leak using radiology and endoscopy is important in order to define the correct indication for EVT and to estimate the expected duration of the treatment. Although EVT has shown very good clinical outcomes, there are some specific contra-indications to consider: malignant perforation, broncho-oesophageal fistula and ischemic conduit necrosis with complete anastomotic dehiscence. Since the device (open-pore sponge attached to a drainage tube connected to a vacuum source) is not complex, it can be self-assembled if not commercially available, and a vacuum redon or bottle can be an alternative to the more sophisticated electronic vacuum pump [79,80]. Lubrification of the device helps to push the sponge through the overtube to its correct position. Endoscopic control of the correct positioning is advised in order to avoid sponge migration and ineffective EVT. Regular replacements should be scheduled at the initiation of EVT for the upcoming weeks.

EVT has shown to be a real asset in the endoscopic management of postoperative digestive defects [20]. However, the technique is prone to several challenges and endeavours, hampering its use in daily clinical practice and depriving critically ill patients of a potentially life-saving treatment. This review aims to provide practical solutions to the aforementioned difficulties. Next to these ‘tips and tricks’ for daily EVT practice, new developments are emerging not only to further improve clinical EVT outcomes, but also to facilitate the labour-intensive EVT procedure. Additionally, the prophylactic or pre-emptive use of EVT in case of high-risk or redo oesophagectomy is under study but has shown conflicting results so far, so further study is awaited [49,84,85]. Case series of successful EVT in young infants using small calibre self-assembled sponge devices are also emerging, illustrating the expanding use and indications of EVT [55,66,86]. One of the latest developments is the hybrid VacStent combining the advantages of EVT and a self-expandable metal stent to treat oesophageal perforation and anastomotic leak (VacStent, Micro-Tech, Düsseldorf, Germany; VacStent, Medech AG, Steinhausen, Switzerland) [87,88]. However, this device is placed inside the oesophageal lumen, not into the extraluminal cavity, and it does not require replacement every 3 to 5 days. Further study is required to define its optimal indications and to compare it with EVT and other endoscopic techniques such as stenting, clips and suturing.

## 8. Core Tip

Endoscopic vacuum therapy (EVT) is a new, safe and effective technique to treat anastomotic leaks and perforations in the upper and lower gastrointestinal tract. Although EVT has shown very good clinical outcomes, the procedure may be difficult to perform, hampering its use in daily clinical practice and depriving critically ill patients of a potentially life-saving therapy. The current review highlights the possible hurdles of EVT and provides practical solutions to facilitate its use in daily clinical practice.

## Figures and Tables

**Table 1:** Literature overview of case series on upper gastrointestinal endoscopic vacuum therapy.

Year	Author	N Patients	N Sponges	Clinical Success	Adverse Events	Upper GI Tract Defect	Ref.
2008	Wedemeyer J	2	5	100%	No	Postoperative leaks	[23]
2010	Loske G	10	1–7	100%	1 sponge rupture	Various defects	[29]
2010	Loske G	1	2	100%	No	Boerhaave syndrome	[30]
2010	Ahrens M	5	8–12	100%	2 strictures 1 death (aortic fistula)	Postoperative leaks	[31]
2010	Weidenhagen R	6	5–14	100%	1 stricture	Postoperative leaks	[32]
2011	Loske G	14	1–10	93%	2 sponge migrations 1 sponge rupture 1 stricture	Various defects	[33]
2012	Kuehn F	9	1–13	89%	1 death (sepsis)	Various defects	[34]
2013	Schorsch T	24	1–12	96%	1 stricture	Various defects	[35]
2013	Schniewind B	17	NA	88%	2 deaths	Postoperative leaks	[36]
2013	Brangewitz M	32	5–28	84%	1 bleeding 1 sponge rupture 1 bronchial fistula 3 strictures 5 deaths	Various defects	[37]
2013	Lenzen H	3	5–12	100%	No	Postoperative leaks	[38]
2014	Bludau M	14	1–9	86%	2 deaths	Various defects	[39]
2014	Heits N	10	2–12	90%	1 death (cardiac failure)	Non-surgical defects	[40]
2014	Schorsch T	35	1–21	91%	1 sponge rupture 1 death	Various defects	[41]
2015	Lee HJ	1	6	100%	1 stricture	Postoperative bronchial fistula	[42]
2015	Mennigen R	22	1–18	86%	3 deaths (cardiac failure, pneumonia)	Postoperative leaks	[43]
2015	Loske G	10	1–3	100%	No	Non-surgical defects	[44]
2015	Möschler O	10	1–39	70%	2 deaths (sepsis)	Various defects	[45]
2016	Smallwood NR	6	2–12	100%	No	Non-surgical defects	[46]
2016	Kuehn F	21	1–14	91%	1 stricture 1 death (sepsis)	Various defects	[47]
2017	Laukoetter MG	52	1–25	94%	4 strictures 2 deaths (EVT-related bleeding)	Various defects	[48]
2017	Neumann PA	8	2–11	75%	3 strictures	Pre-emptive EVT	[49]
2018	Bludau M	77	1–9	78%	10 deaths (sepsis, bleeding, embolism)	Various defects	[50]
2018	Pournaras DJ	21	3–12	95%	2 bleedings 1 death (sepsis)	Various defects	[51]
2018	Heits N	23	NA	91%	2 sepsis 6 strictures	Postoperative leaks	[52]
2018	Noh SM	12	1–6	67%	1 bleeding 1 stricture 1 death (aspiration pneumonia)	Postoperative leaks	[53]
2018	Still S	13	4–6	92%	1 death (palliation)	Various defects	[54]
2018	Manfredi MA	17	1–3	88%	1 increased-size perforation	Various defects (paediatric)	[55]
2019	Berlth F	34	1–9	86%	1 stricture	Postoperative leaks	[56]
2019	Min YW	20	2–12	95%	7 strictures 1 death (palliation)	Postoperative leaks	[57]
2019	Alakkari A	2	6–13	100%	No	Various defects	[58]
2020	Sendino O	11	7	91%	3 strictures 1 death (sepsis)	Various defects	[59]
2020	Oude Nijhuis RA	2	1	100%	No	Pneumatic dilatation achalasia	[60]
2020	Rubicondo C	2	5–6	100%	No	Postoperative leaks	[61]
2021	Jung CFM	30	1–13	73%	2 bleedings 2 deaths (sepsis)	Various defects	[62]
2021	De Pasqual CA	8	5–14	63%	1 bleeding	Postoperative leaks	[63]
2021	Zhang CC	55	1–14	89%	1 bleeding 4 deaths (sepsis, cardiac arrest)	Postoperative leaks	[64]
2021	Book T	116	NA	79%	10 deaths	Various defects	[65]
2021	Ritz LA	4	NA	75%	2 strictures	Various defects (paediatric)	[66]
2022	Mastoridis S	7	1–4	86%	3 strictures 1 death (sepsis)	Various defects	[67]
2022	El-Sourani N	13	4–18	92%	No	Postoperative leaks	[68]
2022	Markus A	20	5–7	90%	1 death (cardiac arrest)	Postoperative leaks (bariatric)	[25]
2022	Richter F	102	1–37	86%	5 bleedings 10 strictures 7 deaths	Various defects	[69]
2022	Stathopoulos P	10	2–3	100%	No	Non-surgical defects	[70]
2022	Reimer S	156	2–8	85%	3 bleedings 9 bronchial fistula 17 strictures	Various defects	[24]
2023	Chon SH	20	1–6	75%	No	Postoperative leaks	[71]
2023	Maier J	17	2–12	71%	7 strictures	Postoperative leaks	[72]
2023	Panneerselvam K	20	1–12	80%	No	Various defects	[73]

## Supplementary Materials

The following supporting information can be downloaded at: https://www.mdpi.com/article/10.3390/life13061412/s1, Video S1: Procedure of EVT in a patient with anastomotic leak after oesophagectomy.
